# Screening for AMPA receptor auxiliary subunit specific modulators

**DOI:** 10.1371/journal.pone.0174742

**Published:** 2017-03-30

**Authors:** Caleigh M. Azumaya, Emily L. Days, Paige N. Vinson, Shaun Stauffer, Gary Sulikowski, C. David Weaver, Terunaga Nakagawa

**Affiliations:** 1 Department of Molecular Physiology and Biophysics, Vanderbilt University School of Medicine, Nashville, Tennessee, United States of America; 2 Vanderbilt Institute of Chemical Biology High Throughput Screening Core, Vanderbilt University School of Medicine, Nashville, Tennessee, United States of America; 3 Department of Chemistry, Vanderbilt University, Nashville, Tennessee, United States of America; 4 Department of Pharmacology, Vanderbilt University School of Medicine, Nashville, Tennessee, United States of America; 5 Center for Structural Biology, Vanderbilt University School of Medicine, Nashville, Tennessee, United States of America; 6 Vanderbilt Brain Institute, Vanderbilt University School of Medicine, Nashville, Tennessee, United States of America; Bilkent University, TURKEY

## Abstract

AMPA receptors (AMPAR) are ligand gated ion channels critical for synaptic transmission and plasticity. Their dysfunction is implicated in a variety of psychiatric and neurological diseases ranging from major depressive disorder to amyotrophic lateral sclerosis. Attempting to potentiate or depress AMPAR activity is an inherently difficult balancing act between effective treatments and debilitating side effects. A newly explored strategy to target subsets of AMPARs in the central nervous system is to identify compounds that affect specific AMPAR-auxiliary subunit complexes. This exploits diverse spatio-temporal expression patterns of known AMPAR auxiliary subunits, providing means for designing brain region-selective compounds. Here we report a high-throughput screening-based pipeline that can identify compounds that are selective for GluA2-CNIH3 and GluA2-stargazin complexes. These compounds will help us build upon the growing library of AMPAR-auxiliary subunit specific inhibitors, which have thus far all been targeted to TARP *γ*-8. We used a cell-based assay combined with a voltage-sensitive dye (VSD) to identify changes in glutamate-gated cation flow across the membranes of HEK cells co-expressing GluA2 and an auxiliary subunit. We then used a calcium flux assay to further validate hits picked from the VSD assay. VU0612951 and VU0627849 are candidate compounds from the initial screen that were identified as negative and positive allosteric modulators (NAM and PAM), respectively. They both have lower IC_50_/EC_50_s on complexes containing stargazin and CNIH3 than GSG1L or the AMPAR alone. We have also identified a candidate compound, VU0539491, that has NAM activity in GluA2(R)-CNIH3 and GluA2(Q) complexes and PAM activity in GluA2(Q)-GSG1L complexes.

## Introduction

AMPA-type ionotropic glutamate receptors (AMPARs) are critical for excitatory synaptic transmission and their impairment negatively impacts cognition, mood, and behavior. Being able to alter their function, or dysfunction, is thus integral to understanding the physiology of neurons and our ability to treat various neurological diseases [1–3]. Negative and positive allosteric modulators (NAMs and PAMs) targeting the channel forming subunits of AMPARs have been developed as potential therapeutics. For example, NAMs, such as perampanel [4], have been used to attenuate seizures in epileptic patients while PAMs, such as CX-516 [5], have been shown to have anti-depressant effects. Although these compounds are effective, they can have severe side effects including dizziness and motor impairment [6, 7].

Each AMPAR subunit is composed of an N-terminal domain (NTD), ligand binding domain (LBD), transmembrane (TM) helices, and C-terminal domain (CTD). The core unit of function for AMPARs is the pore forming tetramer that acts as a ligand gated ion channel, allowing for depolarization of the postsynaptic neuron upon the binding of neurotransmitter glutamate [8]. Mature homo or heterotetramers, assembled from GluA1-GluA4 subunits, have diverse functions due to various factors, such as subunit composition [9], alternative splicing [10], RNA editing [11], posttranslational modifications [12], and association with certain auxiliary subunits [13–20].

The *in vivo* importance of AMPAR auxiliary subunits has become clear from extensive electrophysiological, proteomic, and mutational studies [21, 22]. The highly divergent structures of auxiliary subunits parallel their broad spectrum of functional modulation of AMPARs. It is, therefore, conceivable that specifically targeting AMPAR-auxiliary subunit complexes would enable a variety of functional consequences, some of which may be useful for therapeutics. Selectively targeting specific AMPAR-auxiliary subunit complexes with drugs would be highly beneficial in the clinic and may enable us to determine which type of AMPAR-auxiliary subunit complex is responsible for specific disease phenotypes. NAMs have been identified to target TARP γ-8 containing AMPARs [23, 24]. Here, we report a high-throughput screening (HTS) pipeline that allowed us to obtain new candidate NAMs and PAMs that act preferentially on defined AMPAR-auxiliary subunit complexes.

We chose to screen for compounds that act on three auxiliary subunits that modulate AMPAR function differently. The auxiliary subunits studied in this screen are TARP *γ*-2 (stargazin), cornichon homolog 3 (CNIH3), and germline specific gene 1 like protein (GSG1L). They are expressed in different but partially overlapping neuronal populations in the CNS and provide an opportunity to identify chemical compounds that could serve as brain region-selective AMPAR modulators. Stargazin is concentrated in the cerebellar granule cells, CNIH3 is enriched in the hippocampus and cortex, and GSG1L is expressed in the striatum and cortex. Stargazin and CNIH3 are both positive regulators of AMPAR gating kinetics [14, 18] and GSG1L suppresses AMPAR activity [25, 26].

To identify compounds that target the AMPAR-stargazin and AMPAR-CNIH3, specifically, we developed a high-throughput cellular assay using a voltage-sensitive dye (VSD) that shows an increase in fluorescence proportional to membrane depolarization. Identified hits were then filtered by a series of counter-screens to eliminate false positives and to determine specificity. Finally, a calcium flux assay using the calcium permeable isoform of GluA2, which is not RNA edited at the critical pore-lining amino acid [27], was performed to further characterize the hit compounds. These assays identified a NAM with higher potency on AMPAR complexes containing stargazin and CNIH3, a PAM that reproduces our VSD assay finding of auxiliary subunit dependent activity in electrophysiology, and a compound with PAM or NAM activity depending on which auxiliary subunits are present. These experiments have proven to be an effective way to identify candidate compounds as AMPAR auxiliary subunit specific PAMs and NAMs and could easily be applied to KAR-Neto1/2 and NMDAR-Neto1 complexes as well as non-iGluR-auxiliary subunit complexes worth investigating as therapeutic targets.

## Methods

### Chemicals and media

DMEM (Corning), FBS (Atlanta Biologicals), PenStrep (Gibco), NBQX (Vanderbilt Chemical Synthesis Core), sodium butyrate (Sigma), doxycycline (Clontech), HBSS (Gibco), HEPES (Sigma), FLIPR Membrane Potential Assay Kit (Molecular Devices), glutamate (Fisher), probenecid (Fisher), NaCl (Sigma), KCl (Sigma), MgCl_2_ (Sigma), CaCl_2_ (Sigma), glucose (Sigma), cyclothiazide (Tocris), CX-546 (Tocris), and fluorowillardiine (Tocris).

### Cell lines

Cell lines used in the VSD assays were TetON HEK293 cells. This was the parental cell line for all stable cell lines created. We used GluA2flip(R) TetON HEK cell clone #4 that doxycycline (DOX) dependently expresses GluA2flip(R) (A2R). The A2R cells were used as the base cell line to derive cell lines that co-express auxiliary subunits. Specifically, we generated A2R cells that constitutively express pBOSS-stg-IRES-mCherry clone #7 (A2R-stg) and A2R cells that constitutively express CNIH3 clone #3–3 (A2R-C3). Cell lines used in the calcium flux assay were GluA2flip(Q)-FLAG + GSG1L-1D4 pTREt-Va dual expression in TetON HEK cell clone #20 (A2Q-GSG), GluA2flip(Q)-FLAG + CNIH3-1D4 pTREt-Va dual expression in TetON HEK cell clone #8 (A2Q-C3), GluA2flip(Q)-FLAG pTREt-Va in TetON HEK cell clone #5 (A2Q), and tethered GluA2flip(Q)-FLAG-stargazin [28] in pTREt-Va dual expression in TetON HEK cell clone #13 (A2Q-stg) (Summarized in Table 1).

**Table 1:** Summary of cell lines used for screening.

Cell Lines	Abbrev.	Description
TetON HEK cell	TetON	parental cell line
TetONGluA2flip(R) clone #4	A2R	DOX dependent A2R
TetONGluA2flip(R) clone #4 pBOSS-CNIH3 clone #3–3	A2R-C3	DOX dependent A2R, constitutive C3
TetONGluA2flip(R) clone #4 pBOSS-stg-IRES-mCherry clone #7	A2R-stg	DOX dependent A2R, constitutive C3
GluA2flip(Q)-FLAG + GSG1L-1D4 pTREt-Va TetON clone #20	A2Q-GSG	DOX dependent A2Q and GSG
GluA2flip(Q)-FLAG + CNIH3-1D4 pTREt-Va TetON clone #8	A2Q-C3	DOX dependent A2Q and C3
GluA2flip(Q)-FLAG pTREt-Va TetON clone #5	A2Q	DOX dependent A2Q
GluA2flip(Q)-FLAG-stargazin pTREt-Va TetON clone #13	A2Q-stg	DOX dependent A2Q and stg, tethered

The stable HEK cell lines that were used for all screening in this paper, together with each assigned abbreviation and doxycycline (DOX) dependency of protein expression.

### Vanderbilt Discovery Library (VDL)

The Vanderbilt Discovery Collection is a library of 100,000 compounds that have been curated by the Vanderbilt Institute of Chemical Biology’s High Throughput Screening facility for screening in biological systems to maximize lead potential and diversity (http://www.vanderbilt.edu/hts/services.html).

### Voltage-Sensitive Dye (VSD) screening assay

Initial screening was performed on 39,202 compounds. Compound solutions were prepared as fresh aliquots by transferring 150 nl of selected compounds from VDL plates into Greiner 384-pp round-bottom plates using an Echo 555 (Labcyte) and diluted in 30 μl 1X FLIPR Blue VSD dye (Molecular Devices, cat #R8034) to 50 μM using a Combi liquid dispenser (Thermo). Greiner 384-pp round-bottom plates containing glutamate for the second add were dispensed by hand using a multi-channel pipet at 5X final concentration. A2R-stg cells were plated in 384-well BD PureCoat amine-coated plates (Corning Life Sciences) at 16k cells/well in DMEM medium supplemented with 10% heat-inactivated FBS and 100U/mL PenStrep antibiotic. NBQX, sodium butyrate, and DOX were added to the cells at 30 μM, 1 mM, and 8 μg/ml, respectively. Cells were incubated overnight (O/N) and washed 4 times in HBSS containing 20 mM HEPES, pH 7.4, on an ELX405CW liquid aspirator and dispenser (BioTek). Buffer was left in the wells and an equal amount of 2X FLIPR dye solution was added and incubated on the cells at room temperature for 45 min. Fluorescence signal was collected at 1 Hz using Ex. 480±20 nm/Em. 540±40 nm on a Hamamatsu Functional Drug Screening System 6000 (FDSS). Baseline signal was collected for 10 seconds followed by addition of 10 μl of 5X compound for a final concentration of 10 μM. After 290 seconds, 12 μl of a 5X glutamate solution was added resulting in a concentration approximately 50% of the maximally effective glutamate concentration (EC_50_).

Hits were selected from this initial screen using 4 different criteria, which we termed CMPDslope, CMPDmaxmin, GLUslope, and GLUmaxmin. CMPDslope was measured in a 10 sec window following the initial compound addition and CMPDmaxmin is the difference in the maximal and minimal fluorescence values found in the 100 sec window following compound addition. GLUslope is the fitted slope of the increase in fluorescence within 10 sec after adding glutamate. GLUmaxmin is the difference in the minimum and maximum value in fluorescence signal reached in the 100 sec window after adding glutamate. Compounds were classified as hits if they differed by 3 standard deviations of the mean for each criterion. Tier 1 hits were classified as those that hit in the GLUslope, GLUmaxmin, and CMPDmaxmin windows. Tier 2 hits were classified as compounds that hit in the GLUslope window and either the GLUmaxmin or CMPDmaxmin window. Tier 3 hits were classified as those that hit in only the GLUslope or the GLUmaxmin window. Tier 4 hits were classified as compounds that hit only in the CMPD window, CMPDslope or CMPDmaxmin. Hit selection was further narrowed using the criterion that their signal must return to near baseline values before entering the glutamate add window.

### VSD counter-screens

Hits were first counter-screened against A2R cells. The same protocol as the initial screen was used for the counter-screen and compounds that were found to be stargazin specific were further counter-screened against parental TetON HEK293 cells in the same way to see if their observed activity was due to receptors endogenous to HEK cells. Compounds that did not hit on either A2R or TetON cells were screened against A2R-C3 cells to determine whether they were stargazin or auxiliary subunit specific.

*VSD concentration response curves (CRCs*): After the above counter-screens, compounds that remained positive were moved forward to collect complete CRCs for A2R-stg and A2R-C3 cell lines. This assay was carried out in the same way as the VSD screening assay except a 10-point concentration curve, from 40 μM to 10 nM, was plated in triplicate for each compound on a single 384-well plate. Data were summarized by plotting them as %max GLUslope against log [compound] that was fit to a four-parameter logistical model. %max GLUslope is defined as a normalized GLUslope expressed as a percentage of the mean maximum GLUslope, defined as 100, where background mean vehicle control (VHLslope) were subtracted from both values on a per plate basis. Thus, %max GLU slope = (GLUslope-mean VHLslope/mean maxGLUslope-mean VHLslope). Compounds with measureable potency under 10 μM that fit well to a CRC curve for either cell line were reordered as dry samples and another compound CRC in our VSD assay was run to verify that the EC_50_ results could be repeated.

### Calcium flux assays

Compounds that were selected as hits using the VSD assay were subsequently screened against the calcium permeable A2Q-stg and A2Q-C3 cell lines in a glutamate potency fold-shift assay using the calcium sensing dye Fluo-8 (AAT Bioquest cat #21080). This assay is used to measure how pretreatment with compounds shifts the EC_50_ of glutamate on A2Q-stg and A2Q-C3 cells. 30 μM of each compound was pre-incubated with the cells for 120 secs and then an 11-point glutamate CRC from 4 mM to 10 pM was applied to the cells for 180 sec. 250 nl of selected compounds were plated from the reordered compound plates into Greiner 384-PP round-bottom plates using an Echo 555 (Labcyte) and diluted in 40 μl low calcium buffer (10 mM HEPES, pH 7.4, 140 mM NaCl, 5 mM KCl, 1 mM MgCl_2_, 0.5 mM CaCl_2_, and 10 mM glucose) to 2X final concentration using a Combi (Thermo). Greiner 384-pp round-bottom plates containing glutamate solutions were dispensed by hand using a multi-channel pipet at 5X final concentration. Cells were plated in 384-well BD PureCoat amine-coated plates (Greiner) at 10k cells/well in DMEM medium supplemented with 10% heat-inactivated FBS, 100U/mL PenStrep antibiotic, and 30 μM NBQX 40 hours before screening. Sodium butyrate and DOX were added to the cells at 1 mM and 5 μg/mL, respectively, 24 hours before screening. A2Q-stg cells were induced with 10 μg/mL DOX. Cells were incubated O/N and washed 4 times in low calcium buffer on an ELX405CW liquid aspirator and dispenser (BioTek). Buffer was left in the wells and an equal amount of 4.6 μM (2X) Fluo-8 (AAT Bioquest cat #21080) solution with 2.5 mM probenecid was added and incubated on the cells at room temperature for 40 minutes. Probenecid was added to block dye efflux from the cells [29]. Dye was washed off with low calcium buffer using the ELX405CW (BioTek) and plates were immediately inserted into the FDSS (Hamamatsu). Fluorescence signal was collected at 1 Hz using Ex. 480±20 nm/Em. 540±40 nm. Baseline signal was collected for 10 seconds followed by addition of 20 μl of 2X compound for a final concentration of 30 μM. After 290 seconds, 10 μl of a 5X glutamate solution in high calcium buffer (10 mM HEPES, pH 7.4, 140 mM NaCl, 5 mM KCl, 1 mM MgCl_2_, 9 mM CaCl_2_, and 10 mM glucose) was added for 180 seconds.

For compounds that showed activity in the glutamate potency fold-shift assay, CRCs were run against the calcium permeable A2Q-stg, A2Q-C3, A2Q-GSG1L, and A2Q cell lines using the same FDDS addition protocol as above for a compound concentration range of 30 μM to 30 nM. These compound CRCs were applied to the cells 2 minutes before a 1mM glutamate stimulation. Additional replicate plates of compound followed by EC_50_ of glutamate were evaluated, both controlled with CTZ curves (data not shown). Normalized FDSS traces were curve fit to CRCs using the initial GLUslope (GLUslope1) in a 3–6 sec window after glutamate application. CRCs were also plotted for the traces’ area under the curve (AUC) in the GLUmaxmin window. Full CTZ and NBQX curves were used as reproducibility controls on each plate. Compounds were plotted as %max GLUslope against log [compound] and fit to a four-parameter logistical model as in the VSD.

### Electrophysiology

A2R-stg cells were plated on HNO_3_ washed coverslips coated with 1:20000 poly-d-lysine (incubated for 20 min, washed with PBS 2x and media 1x) for 2 hours. They were induced with 5 μg/mL DOX for 24 hours before recording. A2R cells were plated on 1:60000 poly-d-lysine for 2 hours and induced with 7.5 μg/mL DOX for 30 hours before recording. The cells were lifted from the coverslip after whole cell configuration was achieved and brought in front of the theta tubing. Ligand (1mM glutamate) was applied to the cells via theta tubing glass capillary mounted on a piezo actuator (P-830.30, Physik Instrumente) controlled by an LVPZT amplifier (E-505, Physik Instrumente), DAQ device (NI USB-6221, National Instruments), and LabView software (National Instruments). Recording was done using a single channel of a Multiclamp700B Amplifier (Axon Instruments) operated by pClamp10 software. Signals were digitized using Digidata1440A (Axon Instruments) at a sampling rate of 50 kHz and low pass filtered at 2kHz. Borosilicate glass capillaries (O.D. 1.5 mm, I.D. 0.86 mm, Sutter) were pulled to manufacture electrodes with pipette resistances of 3.5–5 MΩ.

Internal solution was (in mM) 110 NaCl, 10 NaF, 5 EGTA, 0.5 CaC_l2_, 1 MgCl_2_, 10 Na_2_ATP, 5 HEPES, adjusted to pH 7.3 with CsOH and 295 mOsm. External solution was (in mM) 145 NaCl, 2.5 KCl, 1.8 CaCl_2_, 1 MgCl_2_, 5 HEPES, 10 glucose, adjusted to pH 7.3 with NaOH and 301 mOsm. Standard solution without ligand was the external solution. The ligand solution contained 1mM glutamate in external solution, supplemented with 2mM glucose and 3mM NaCl to facilitate visualizing the interface of the two solutions and recording liquid junction potential after breaking the patch. The 10–90% rise time of liquid junction potential was around 300μsec. VU0627849 was dissolved in external solution containing 1 mM glutamate to a final concentration of 40 μM. Tips of the tubing for each solution were positioned immediately before the opening of one compartment of the theta tubing. Drug-containing and drug-free glutamate solutions were switched using a manual valve located between the solution reservoir and theta glass tube. Solution speed was adjusted by the height of the reservoir using gravity.

## Results

### Cell lines for voltage-sensitive dye (VSD) assay

Cell based assays in combination with HTS were used to identify compounds that specifically target the AMPAR in complex with the TARP *γ*-8 auxiliary subunit [23, 24]. Taking an analogous approach, we generated multiple cell lines as summarized in Table 1 and developed a VSD based cellular assay compatible with HTS to screen ~39,000 compounds from the Vanderbilt Discovery Library (VDL). In detail, we first made HEK cell lines that constitutively expressed an auxiliary subunit and doxycycline (DOX) dependently expressed the GluA2 subunit of the AMPAR. For these cells the flip splice isoform and pore RNA-edited (i.e. arginine (R) in the edited site) form of GluA2 was used, resulting in a cyclothiazide (CTZ)-sensitive and calcium-impermeable channel. The constitutive expression of auxiliary subunits ensured an excess of auxiliary subunits to associate with mature AMPARs. The RNA-edited form of GluA2, with an arginine in the pore, naturally conducts less current than the unedited Q isoform of the channel, keeping these cell lines healthier during the assay. Nevertheless, while maintaining these lines, cells were cultured in 30 μM NBQX, an AMPAR antagonist, to decrease cell death due to excitotoxicity. A2R-GSG and A2R cells did not show any activity in the VSD assay when exposed to glutamate (data not shown), consistent with negative modulatory function of GSG1L and low channel conductance of GluA2flip(R) variant [25, 30].

### A VSD assay screen

**Fig 1:**
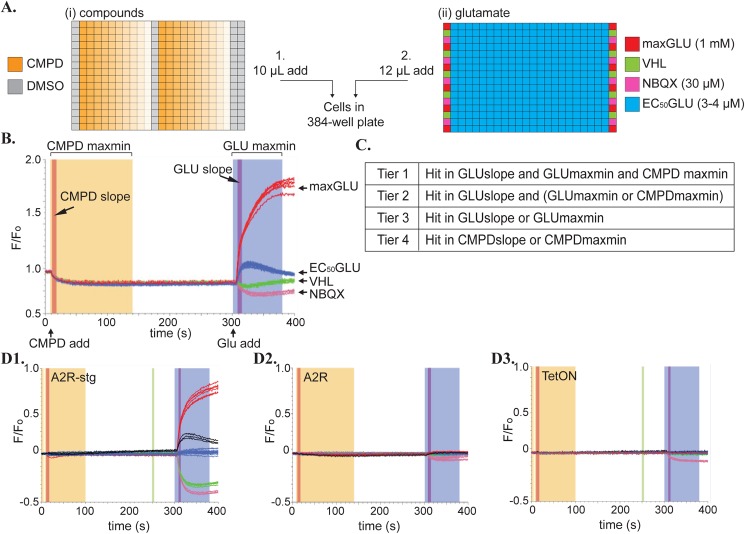
Configuration of VSD assays. **(A)** Arrangement of wells in compound and glutamate plates added to cells by the FDSS. **(i)** Compounds (orange) are added at 10 μM concentration in initial screen and as a 40 μM– 10nM curve (decreasing color saturation) for CRC testing. **(ii)** Controls to determine a Z’ for each plate line the edges of the glutamate plate. Positive control = 1mM glutamate (maxGLU, red), 1X FLIPR Blue dye vehicle (VHL, green), negative control = 30 μM NBQX (NBQX, pink). EC_50_ glutamate (3–4 μM) is added across the plate (EC_50_GLU, blue) with columns 2 and 23 used as EC_50_GLU controls with DMSO. DMSO control was moved to column 12, in CRC plates. **(B)** Normalized fluorescence data (ratio of the F/F_o_) readout for the FDSS on a VSD experiment showing the compound and glutamate additions at 10 sec and 300 sec, respectively. Controls are shown in colors corresponding to their colors in the glutamate plate in A(ii). Different hit windows are shaded in dark orange (CMPDslope), light orange (CMPDmaxmin), purple (GLUslope), and violet (GLUmaxmin). **(C)** Definition of Tier 1–4 hits in our initial screen. Hits were determined as those compounds that deviated from the mean of the test population EC_50_GLU by more than three standard deviations in the windows specified on a per plate basis. **(D)** Example of a compound (black trace) that hit on **(1)** A2R-stg cells but not on **(2)** A2R or **(3)** TetON cells. Controls are maxGLU (red), vehicle (green), 30 μM NBQX (pink), all normalized to EC_50_GLU (blue).

Our initial screening technique utilized a VSD whose fluorescence increases when the cell depolarizes. This allowed us to detect depolarization of HEK293 cell membranes when functional AMPAR complexes were present and gated by glutamate. After pre-incubation with the compounds diluted in VSD dye (Fig 1Ai), glutamate was added to the cells (Fig 1Aii), opening the AMPAR, and resulting in an increase in fluorescent signal. To analyze the results, we used 4 different parameters, which we termed CMPDslope, CMPDmaxmin, GLUslope, and GLUmaxmin, which are described in the methods section above (Fig 1B).

Compounds were classified as hits if a measurement varied by more than 3 standard deviations from the mean signal of an EC_50_ (3–4 μM) amount of glutamate (Fig 1B(blue)) within the test population of each 384-well plate. Hits were further categorized into Tier 1–4 as described in the methods (Fig 1C). The Z’ was used to assess the reliability of the screen in a high-throughput format. Z’ is the ratio of the difference in standard deviations of positive and negative controls over the difference in their means [31]. Values from 0.5–1 indicate that the response being measured is robust enough to be used as an HTS assay. Each glutamate plate contains a positive control (1 mM, maxGLU) and a negative control (30 μM, NBQX) to calculate the Z’ score. An example of a hit compound is shown in Fig 1D (black traces), where a robust response was detected in A2R-stg cells (Fig 1D1) but not in A2R (Fig 1D2) or TetON (Fig 1D3) cells, which serve as counter-screens.

### Responses to known AMPAR ligands in the VSD assay

**Fig 2:**
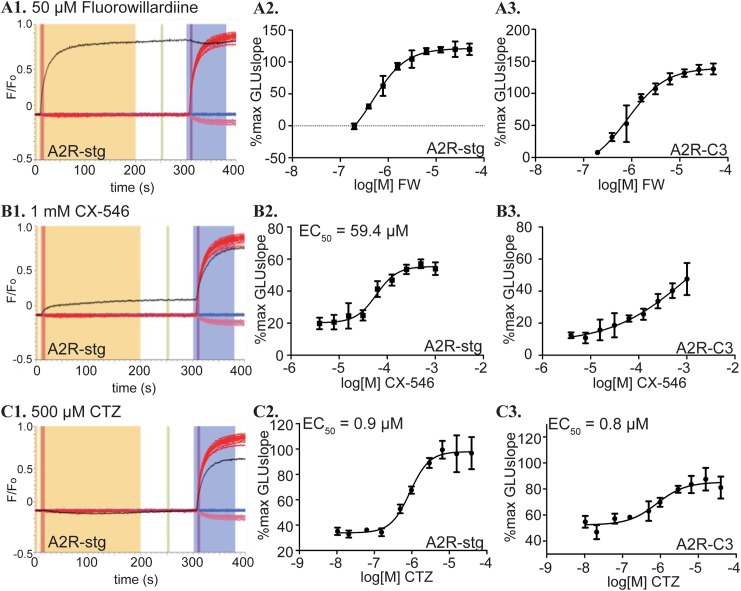
Behavior of established compounds in VSD assay. **(A1)** Normalized fluorescence data for VSD assay on A2R-stg cells with 50 μM fluorowillardiine (black) with maxGLU (red) and NBQX (pink) controls, all normalized to EC_50_GLU (blue). **(A2)** CRC curves for FW against A2-stg and (**A3)** A2-C3 cell lines calculated from the CMPDslope window. %max GLUslope = (CMPDslope—mean VHLslope)/(mean maxGLUslope—mean VHLslope) is further described in methods. (**B1)** Normalized fluorescence data for VSD assay on A2R-stg cells with 1 mM CX-546 (black) with maxGLU (red) and NBQX (pink) controls, all normalized to EC_50_GLU (blue). **(B2)** CRC curves for CX-546 against A2R-stg and (**B3)** A2R-C3 cell lines calculated from the GLUslope window. %max GLUslope = (GLUslope—mean VHLslope)/(mean maxGLUslope—mean VHLslope) **(C1)** Normalized fluorescence data for VSD assay on A2R-stg cells with 500 μM cyclothiazide (CTZ, black) with maxGLU (red) and NBQX (pink), all normalized to EC_50_GLU (blue). **(C2)** CRC curves for CTZ against A2-stg and **(C3)** A2-C3 cell lines calculated from the GLUslope window. Compound EC_50_ values that could be reliably calculated are in the top left corner of each graph.

Known AMPAR ligands were tested against A2R-stg and A2R-C3 cell lines using this method to validate our assay. We tested a partial agonist, fluorowillardiine (FW), and two positive allosteric modulators (PAM), CX-546 and CTZ [32–34]. FW showed a CMPDslope response comparable to the GLUslope response to glutamate alone as expected for a partial agonist (Fig 2A1, compare black FW and red glutamate traces). Due to an incomplete concentration response curve (CRC) (Fig 2A2-3), we were unable to calculate a reliable EC_50_. CX-546 is an ampakine PAM of AMPARs. In the absence of glutamate, there was a slight increase in fluorescence with CX-546 only at high concentrations (>250 μM) (Fig 2B1), which would be detected as a hit even with filtering criteria that we imposed in the actual screening. We found the EC_50_ for CX-546 on our A2R-stg cell line to be 59.4 μM. This is an order of magnitude more potent than the previously reported EC_50_ of 563 μM for GluA2-stargazin [35]. While an EC_50_ could not be accurately calculated for A2R-C3, CX-546 is over two orders of magnitude less potent than for A2R-stg (Fig 2B2-3). CTZ showed the least amount of activity in the compound only window (Fig 2C1), consistent with CTZ being a non-competitive allosteric modulator. The EC_50_s of CTZ on A2R-stg and A2R-C3 cells were similar, 0.9 μM and 0.8 μM, respectively (Fig 2C2-3). These values were closer to published values of 2.2 μM on GluA2 alone [36] and 2 μM of GluA1-stargazin [37] than the EC_50_ value of CX-546. Responses to a known AMPAR partial agonist and PAMs demonstrate that our VSD assay can detect these drugs as hits.

### Screening workflow

**Fig 3:**
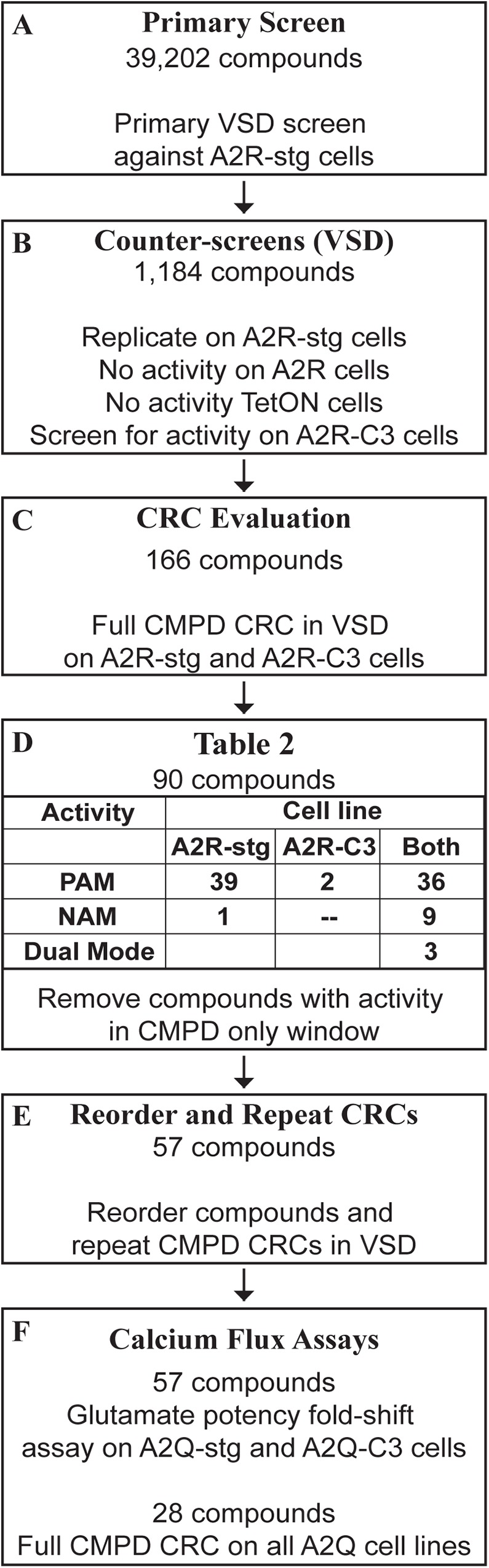
Workflow for identifying AMPAR-auxiliary subunit modulators. **(A)** 39,202 compounds were initially screened using the VSD assay against A2R-stg cells. **(B)** 1,184 hits from (A) were counter-screened against A2R, TetON, and A2R-C3 cells. **(C)** 116 compounds were identified from counter-screening in (B) as being stargazin or auxiliary subunit specific (i.e. they did not hit on A2R or TetON cells). These were tested for full compound CRCs against A2R-stg and A2R-C3 cells using the VSD assay. These CRCs identified 90 hits that fit to sigmoidal dose response curves with potency under 10 μM. **(D)** We identified 39 stargazin specific PAMs, 2 CNIH3 specific PAMs, and 36 PAMs that had activity in both A2R-stg and A2R-C3 cells. We also found 1 stargazin specific NAM and 9 compounds with NAM activity on both cell lines. Three compounds gave opposite effects in the two cell lines. Hits were discarded for reorder if they showed activity in the compound only window. Hits with activity in the CMPD only windows were discarded. **(E)** 57 of the 90 compounds in (D) were re-screened with new batch samples as compound CRCs in the VSD assay. **(F)** 57 hits were tested in the glutamate potency fold-shift calcium flux assay and 28 were subjected to a full compound CRC calcium flux assay to study their effects using an orthogonal approach.

The screening workflow is summarized in Fig 3. Our primary screen tested 39,202 compounds against A2R-stg cells at a dose of 10 μM to detect changes in response compared to EC_50_ glutamate (Fig 3 Box A). From these, we pulled 187 Tier 1 hits, 325 Tier 2 hits, 1509 Tier 3 hits, and 3270 Tier 4 hits. Tier 3 and 4 hits were discarded if they didn’t return to baseline before the addition of glutamate because their GLUslope values were difficult to compare to glutamate alone, reducing the numbers to be counter-screened to 628 and 44 in Tier 3 and Tier 4, respectively. These 1,184 compounds were subjected to counter-screens using duplicate wells of 10 μM compound to determine their activity against A2R and TetON cells (Fig 1D). Those compounds that showed activity would indicate auxiliary subunit and AMPAR independent effect in A2R and TetON cells, respectively, and were discarded. To determine their specificity for stargazin, these compounds were also screened against A2R-C3 cells. We kept compounds that were stargazin specific and that hit on both stargazin and CNIH3 (auxiliary subunit specific) (Fig 3 Box B). Collectively, these counter-screens reduced the number of GluA2-auxiliary subunit specific hits to 166 compounds. Full CRCs (see methods) were obtained against A2R-stg and A2R-C3 cell lines to determine if these compounds would fit to a dose response curve (Fig 3 Box C).

From these initial CRCs using the Vanderbilt Discovery Library plates, 77 PAMs, 10 NAMs, and 3 compounds with different effects on A2R-stg and A2R-C3 were identified. 39 PAMs were stargazin specific, 2 were CNIH3 specific, and 36 potentiated both cell lines. There were fewer NAMs identified, with only one stargazin specific NAM and 9 NAMs that hit both cell lines. 3 compounds showed opposite activity in the two cell lines (Fig 3 Box D). After discarding compounds with a large amount of activity in the compound only window at concentrations lower than 7.5 μM (Fig 3 Box D), 57 of these hits were supplied as dry samples from Life Chemicals to examine their reproducibility (Fig 3 Box E). When repeating CRCs with the new batch of samples, 48 of the 57 compounds were found to reproducibly show a curve fit in the range tested (n = 2). The 57 reordered compounds were evaluated in the calcium flux assays for further testing as described below (Fig 3 Box F).

### A calcium flux assay to further verify hits

**Fig 4:**
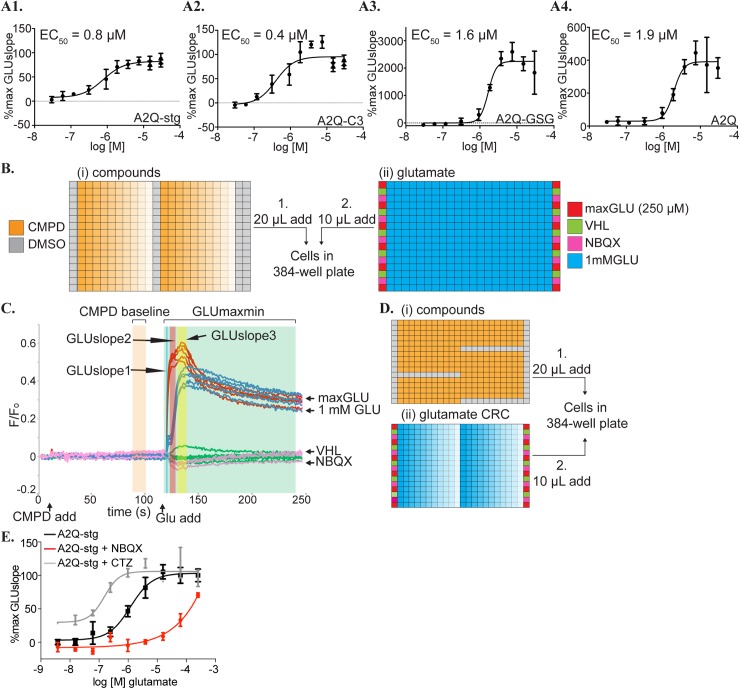
Controls and experimental setup for calcium flux assays. **(A)** CRC curves for CTZ in the presence of 1 mM glutamate from **(1)** A2Q-stg, **(2)** A2Q-C3, **(3)** A2-GSG, **(4)** A2Q cell lines in the calcium flux assay. These CRCs are calculated from the GLUslope1 (t = 122-125s) window. Calculated EC_50_ values are included in the top left of the graph. %max GLUslope = (GLUslope–mean VHLslope) / (mean maxGLUslope–mean VHLslope) as described in methods. **(B)** Compound and glutamate plates added to cells by the FDSS in our Fluo-8 calcium flux compound CRC assay. **(i)** Compounds (orange) are added as a 30 μM– 30 nM CRC (decreasing color saturation). **(ii)** Controls to determine a Z’ for each plate line the edges of the glutamate plates. Positive control = 250 μM glutamate (maxGLU, red), high calcium buffer vehicle (VHL, green), and negative control = 30 μM NBQX (NBQX, pink). 1 mM glutamate is added across the plate (blue) with columns 12 and 23 used as a 1mM glutamate and DMSO control. **(C)** Vehicle subtracted, normalized fluorescence data readout for the FDSS on A2Q-stg cells in a calcium flux experiment showing the compound and glutamate applications at 10s and 120s, respectively. Controls are shown in colors corresponding to their colors in the glutamate plate in (D). Different hit windows are shaded in blue (GLUslope1, 122-125s), red (GLUslope2, 126-132s), yellow (GLUslope3, 140-150s), green (GLUmaxmin). The orange window is a reference baseline (CMPD baseline) prior to the glutamate addition used to determine if the compound shows activity in the absence of glutamate. **(D)** Compound and glutamate plates added to cells by the FDSS in our Fluo-8-based glutamate potency fold-shift assay. **(i)** Compounds (orange) are first added at 30 μM with DMSO controls in columns 1,24, and also in K1-K12, F13-23, P13-23, overlapping with glutamate concentration curves for a per plate comparison to compound. **(ii)** Controls are loaded on the edge as in (Bii) and a glutamate CRC is loaded horizontally ranging from 4 mM to 10 pM (decreasing color saturation). **(E)** An example of the readout for our glutamate potency fold-shift assay. A rightward shift of the NBQX pretreated cells (red) as compared to glutamate alone (black) indicates NAM activity. The leftward shift of CTZ pretreated cells (grey) indicates PAM activity.

Fluo-8 based calcium flux assays were used as a final screen for the 57 ligands selected from the VSD assay and 28 of these showed PAM or NAM activity on A2Q-stg and/or A2Q-C3 cell lines that warranted performing full CRC experiments in all A2Q cell lines (Fig 3 Box F). For this purpose, we had created stable cell lines DOX dependently co-expressing GluA2flip(Q), the pore unedited, calcium-permeable isoform, and each auxiliary subunit (A2Q, A2Q-stg, A2Q-C3, A2Q-GSG; summarized in Table 1). To verify our assay, a compound CRC for CTZ was examined for each cell line, as described below, and gave EC_50_ values ranging from 0.4 to 1.9 μM, which were in good agreement with the known values (2.2 μM on GluA2 alone [36] and 2 μM on GluA1-stargazin [37] (Fig 4A1-4) and similar to those calculated from our VSD assay (Fig 2C2-3).

In brief, the compound CRC assay was conducted as follows (see methods for details). Cells were loaded with Fluo-8 and excess dye was washed out. After 120 secs of pre-incubation with compound at concentrations ranging from 30 μM to 30 nM (Fig 4Bi), 1 mM glutamate was added (Fig 4Bii). In the calcium assay, 250 μM was used as the maxGLU dose because the GLUslope1 values decreased at higher concentrations of glutamate, making it the dose with the maximal GLUslope1 response. This decrease in activity at high glutamate concentration has been seen in other publications [38, 39]. We measured GLUslope in three consecutive time windows, referred to as GLUslope1-3, because three phases were clearly detectable (Fig 4C). The final CRC curves were calculated from GLUslope1, obtained from the first 3–6 sec window.

Prior to compound CRCs, we performed a glutamate potency fold-shift assay to determine how a maximal dose of compound would shift the EC_50_ of glutamate on A2Q-stg and A2Q-C3 cells. In this assay, a maximal dose (30 μM) of each compound (Fig 4Di) was incubated on cells for 120 secs and then a CRC for glutamate ranging from 4 mM to 10 pM was collected (Fig 4Dii). A rightward shift of the CRC for glutamate to a higher EC_50_ indicates NAM activity and a leftward shift to a lower EC_50_ indicates PAM activity (Fig 4E). 28 of the 57 compounds modified the activity, when compared to the glutamate EC_50_ or 1mM glutamate controls per plate. These compounds, considered active, were then evaluated as a CRC of the compounds in the calcium flux assay.

VU compound CRCs were collected against all A2Q containing cell lines using the same protocol as CTZ, described above (Fig 4B). The final compound CRC curves were calculated from GLUslope1. The area under the curve (AUC) in the GLUmaxmin window was used as an alternative measure to calculate the CRC because compounds sometimes deviated from the 1 mM glutamate trace in different GLUslope windows. The AUC measures the accumulation of calcium instead of gating and sometimes produced a CRC fit when GLUslope1 could not derive an EC_50_ value.

### Description of candidate compounds

**Fig 5:**
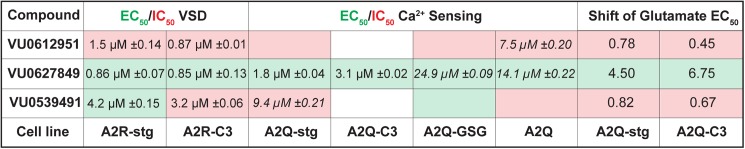
Table of results from VSD and calcium flux assays. EC_50_ or IC_50_ values determined by CRC fits from GLUslope. Boxes highlighted green indicate an EC_50_ or a positive trend, red indicate an IC_50_ or a negative trend. Estimated EC_50_ values are added in italics, but are estimated due to incomplete CRC curves or insufficient differences in %max GLUslope across the CRC. Glutamate potency fold-shift assays indicate how much fold-change occurred in the glutamate EC_50_ when cells were pretreated with 30 μM compound. Values greater than 2 indicate PAM activity and less than 1 indicate NAM activity.

**Fig 6:**
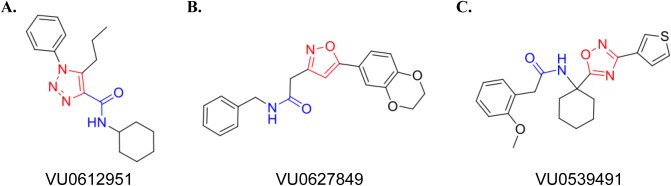
Chemical structures of our candidate hits. **(A)** Structure of VU0612951 highlighting the 1,3-triazole group in red. **(B)** Structure of VU0627849 highlighting the isoxazole group in red. **(C)** Structure of VU0539491 highlighting the 1,2,4-oxadiazole group in red.

As described above, all compounds were subjected to a counter-screen against A2R and TetON cells. While this should, in theory, remove compounds with any activity on AMPAR without auxiliary subunits present, the increased sensitivity of the calcium flux assay revealed that some compounds did show NAM or PAM activity in A2Q cells. Our pipeline has led us to specify 3 compounds for further characterization (summarized in Fig 5) that exhibit the most robust difference in pharmacology between cell lines. In addition, these three compounds have attractive chemical structures and properties for further hit-to-lead exploration. Each of the three molecules identified bears a modular chemical structure with a central five-membered heterocyclic core structure—either a 1,3-triazole, isoxazole, or a 1,2,4-oxadiazole. The trisubstituted triazole containing a carbocyclic amide structure represented by VU0612951 displayed modest NAM activity (Fig 6A), whereas, the disubstituted isoxazole VU0627849 maintains robust potentiator activity (Fig 6B). Lastly, VU0539491, which contains a unique 1,1, disubstituted cyclic structure, also partially related to VU0612951 via the benzylic reverse-amide, shows mixed pharmacology (NAM and PAM) depending on the cell-type (Fig 6C).

**Fig 7:**
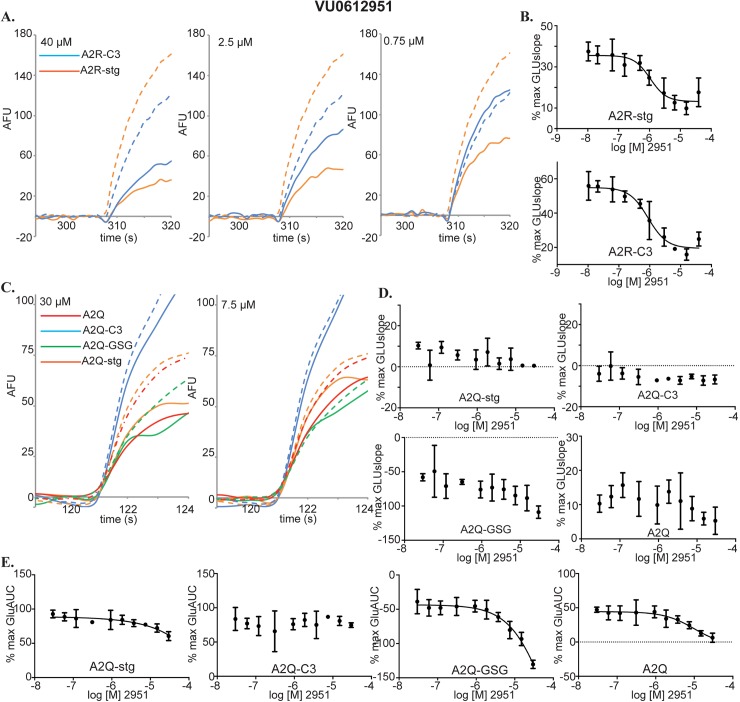
Characterization of VU0612951. **(A)** Raw data for compound CRCs in the VSD assay. A2R-stg (orange) and A2R-C3 (blue). EC_50_GLU traces are represented by dashed lines. Compound concentrations are indicated in the top left corner. **(B)** CRCs calculated from GLUslope in the VSD assay for A2R-stg and A2R-C3 cells. Error bars are standard deviations. **(C)** Raw data for compound CRCs in the calcium flux assay. A2Q (red), A2Q-stg (orange), A2Q-GSG (green), and A2Q-C3 (blue). Dashed lines are signal of 1mM glutamate without compound. **(D)** Compound CRCs in calcium flux assay for A2Q-stg, A2Q-C3, A2Q-GSG, and A2Q cell lines. These are derived from the GLUslope1 window. **(E)** Compound CRCs calculated from the AUC in the GLUmaxmin window of the calcium flux assay plotted as %max AUC in the GLUmaxmin window (see Fig 4C) vs. log [compound].

Hit molecule VU0612951 was identified as a NAM in both A2R-stg and A2R-C3 cell lines in the VSD assay. In the raw VSD traces, NAM activity on A2R-stg was evident even at submicromolar concentrations, while on A2R-C3 cell line the NAM effect required concentrations higher than 1 μM (Fig 7A). The ~40% decreases in %max GLUslope across the CRC (Fig 7B) and right-shifts in the glutamate potency fold-shift assay (Fig 5) indicate strong NAM activity for stargazin and CNIH3 containing AMPAR complexes. The magnitude of effect on GLUslope for VU0612951 was low in the calcium flux assay and only evident at high concentrations (Fig 7C, 30 μM), making it difficult to reliably obtain CRCs. In fact, the CRCs calculated from GLUslope did not show a negative trend on any cell line except A2Q-stg (Fig 7D). We therefore used %max AUC instead of %max GLUslope to derive CRCs (Fig 7E), the results of which are consistent with VU0612951 as a NAM.

**Fig 8:**
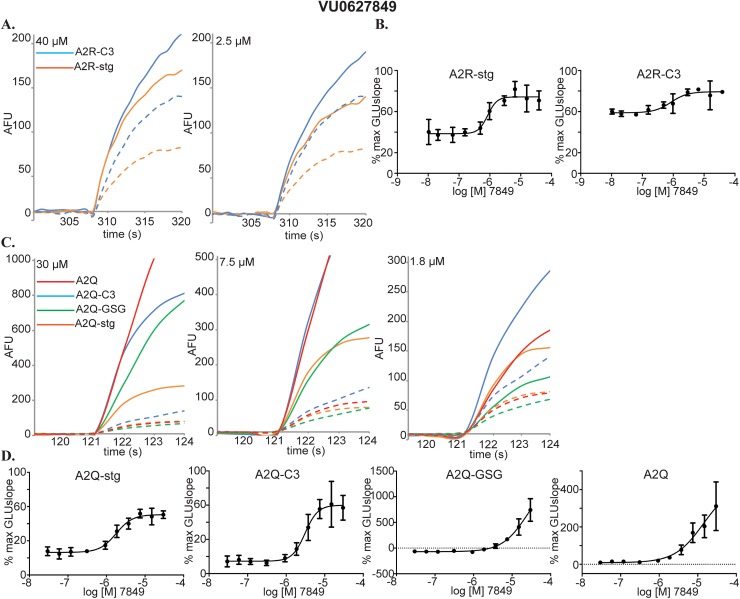
Characterization of VU0627849. **(A)** Raw data for compound CRCs in the VSD assay. A2R-stg (orange) and A2R-C3 (blue). EC_50_GLU traces are represented by dashed lines. Concentrations of compound are indicated in the top left corner. **(B)** CRCs calculated from GLUslope in the VSD assay for A2R-stg and A2R-C3. **(C)** Raw data for compound CRCs in the calcium flux assay. A2Q (red), A2Q-stg (orange), A2Q-GSG (green), and A2Q-C3 (blue). 1 mM glutamate traces are represented by dashed lines. **(D)** CRCs calculated from GLUslope1 in calcium flux assay for A2Q-stg, A2Q-C3, A2Q-GSG, and A2Q cell lines. Plotted as %max GLUslope vs. log [compound].

The most promising PAM discovered was VU0627849. The VSD raw data shows PAM activity on both A2R-stg and A2R-C3 complexes at concentrations as low as 2.5 μM (Fig 8A). These data fit well to CRCs calculated from the GLUslope (Fig 8B). This compound did not show any activity on A2R cells in our counter-screens (Fig 3 Box B). VU0627849 was also a PAM in all GluA2-expressing cell lines in the calcium flux assay. VU627849 potentiated A2Q-stg cells the least compared to all others and its level of potentiation does not change between 7.5 and 30 μM (Fig 8C solid orange). At these concentrations, the increases in response of A2Q-stg cells to VU0627849 were fixed to ~300 arbitrary fluorescence units (AFU), while in the other three cell lines the effects nearly doubled from 7.5 μM to 30 μM. (Fig 8C, compare solid lines between 30 and 7.5 μM). While the increase in GLUslope on A2Q-stg cells fits to a sigmoidal dose response curve, the positive change in %max GLUslope is only ~30% as compared to >80% for the other A2Q containing cell lines. (Fig 8D). VU0627849 shows similar values to CTZ for increase in %max GLUslope across the CRC for A2Q-C3, A2Q-GSG, and A2Q cells. There was no appreciable difference in potency seen between cell lines for CTZ, but VU0627849 shows a lower EC_50_ for A2Q-C3 than A2Q-GSG or A2Q cells (Fig 4A1-4 and Fig 5). Collectively, our data suggest that this compound acts as a PAM only on A2R when in complex with auxiliary subunits, but positively modulates A2Q regardless of the presence of auxiliary subunits. In fact, VU0627849 is least efficacious on the A2Q-stg cells when comparing all four complexes we tested.

**Fig 9:**
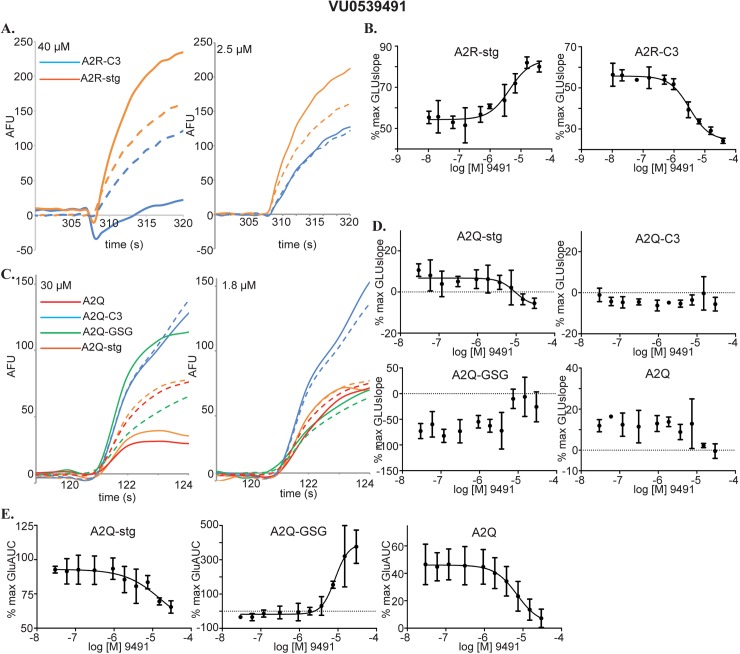
Characterization of VU0539491. **(A)** Raw data for compound CRCs in the VSD assay. A2R-stg (orange) and A2R-C3 (blue). EC_50_GLU traces are represented by dashed lines. Compound concentrations are indicated in the top left corner. **(B)** CRCs calculated from GLUslope in VSD assay for A2R-stg and A2R-C3. **(C)** Raw data for compound CRCs in the calcium flux assay. A2Q (red), A2Q-stg (orange), A2Q-GSG (green), and A2Q-C3 (blue). Traces obtained from applying 1 mM glutamate are represented by dashed lines. **(D)** Compound CRCs calculated from the GLUslope1 window in our calcium flux assay for A2Q-stg, A2Q-C3, A2Q-GSG, and A2Q cell lines. These show negative and positive trends but no curve fits. **(E)** Compound CRCs calculated from the AUC in the GLUmaxmin window of the calcium flux assay plotted as %max AUC vs. log [compound] as in (7E).

VU0539491 was a particularly interesting compound identified by our VSD screen. It acted as a PAM on A2R-stg cells, but a NAM in A2R-C3 cells in the VSD assay (Fig 9A and 9B). While exhibiting a slight decrease in signal in the raw data from the calcium flux assay for A2Q-C3 cells (Fig 9C, 30 μM blue trace), it was clearly categorized as a NAM in the glutamate fold-shift assay (Fig 5). In addition, VU0539491 showed PAM activity in the A2Q-GSG cell line (Fig 9C green trace), which appeared as a positive trend in the CRC calculated from GLUslope (Fig 9D A2Q-GSG) and was corroborated by a more robust fit in the CRC calculated from the AUC (Fig 9E A2Q-GSG). Slight NAM activity on A2Q and A2Q-stg cell lines can be seen in the highest dose (40 μM) of VU0539491 (Fig 9C orange and red traces). As in the A2Q-GSG cells, the CRCs calculated from GLUslope showed negative trends for A2Q and A2Q-stg (Fig 9D A2Q-stg and A2Q), but CRCs calculated from the AUC show more robust curve fits (Fig 9E A2Q-stg and A2Q). The combination of these data indicates that VU0539491 acts as a slight NAM on A2R-C3 and A2Q, but a PAM on A2Q-GSG.

**Fig 10:**
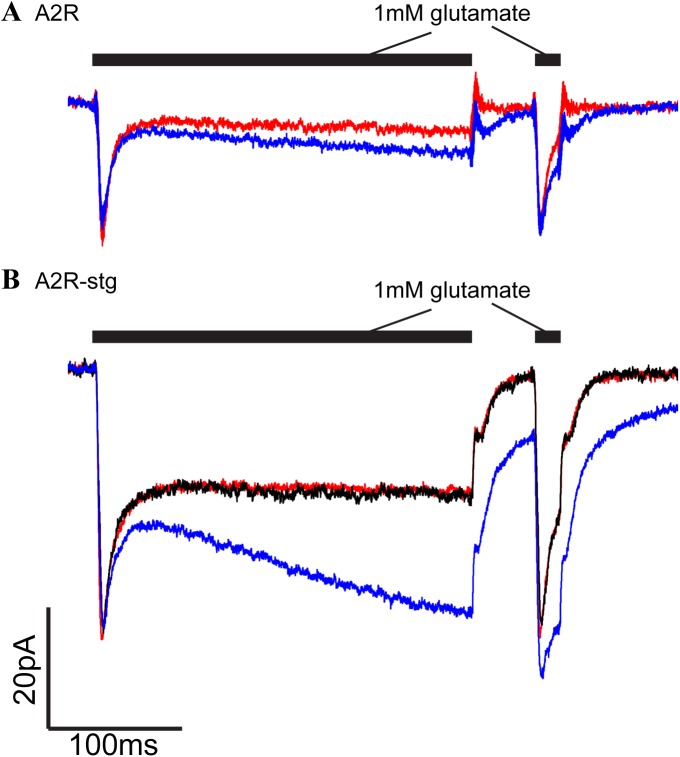
Electrophysiology with VU0627849. **(A**) Whole-cell recordings of A2R cell line with (blue) and without (red) VU0627849 (40μM). **(B)** Whole-cell recordings of A2R-stg cell line with (blue) and without (red) VU0627849 (40μM). Recording after washout of drug is in black. In these experiments, glutamate (1mM) is applied for 100 ms and 20 ms pulses with or without VU0627849.

We have conducted a preliminary electrophysiological investigation of the compound VU0627849. Using a fast ligand application system, A2R and A2R-stg cells were stimulated with 1mM glutamate using a 100 ms pulse followed by a 50 ms interval and a second 20 ms pulse to evaluate recovery from desensitization (Fig 10A and 10B red traces). In the presence of 40 μM VU0627849, we observed no change in peak amplitude but delayed increase in resensitization within 10 ms following initial activation and desensitization (Fig 10B blue trace). In addition, an increased amplitude in the second pulse of glutamate was observed. These effects are drug specific because wash out of VU0627849 restored the original current (Fig 10B, black trace). We also see a small effect on A2R cells that was not seen in the VSD assay (Fig 10A blue trace). VU0627849 acted as a PAM on A2R-stg cells, corroborating our HTS data from the VSD assay.

## Discussion

High-throughput screening has been valuable in identifying TARP *γ*-8 subunit specific NAMs [23, 24, 40]. Given the structural and functional variety of AMPAR auxiliary subunits we predicted that there should be more compounds that are targeted against specific members of this family of complexes and chose to study those containing stargazin, CNIH3, and GSG1L. From a relatively small library of 39,000 compounds, our HTS workflow identified 3 compounds for further studies.

In the current study, we focus on the PAMs and NAMs that showed activity in the presence of glutamate. Our initial data already identified compounds that show activity in the absence of glutamate and there remains a possibility of identifying new agonists by choosing to study compounds that hit in the CMPDslope window, as seen when testing fluorowillardiine. Furthermore, the primary screening was conducted for only one third of the existing Vanderbilt Discovery Library. Collectively, our HTS workflow described here has potential to identify additional candidate compounds in the future.

An interesting byproduct of our study is the pharmacology of CX-546 on AMPARs in complex with different auxiliary subunits. CX-546, an ampakine PAM, shows more than an order of magnitude increase in potency on A2R-stg cells over A2R-C3 cells. In a previous report, CX-546 had different E_max_ values when applied to AMPAR with different TARPs, but the difference in CX-546 EC_50_ values for different Type I TARPs were all within the same order of magnitude [35]. While absolute potencies in our VSD assay may not be reliable, we postulate that the relative difference in the potencies of CX-546 on the two complexes indicates unique pharmacology dependent on auxiliary subunits.

We identified 3 compounds for future characterization. We propose that VU0612951 is an indiscriminate NAM. While we were aiming to identify compounds with subunit specific effects, this compound has less standard deviation and a more robust response from A2R-stg cells than any others. The overall NAM profile of VU0612951 and its modular triazole core will make this an attractive template for further chemical modification to identify analogs with subunit specificity (Fig 6A). VU0627849 is an effective PAM that responds in the VSD and calcium flux assays similarly to CTZ, though they have dissimilar structures (Fig 6B). Unfortunately, it also hits multiple AMPAR complexes, but it has very little activity on A2R cells and seems to affect A2Q-stg cells less than the other A2Q cell lines in both calcium flux assays. VU0627849 could be used as a starting scaffold to determine if functional groups could be added to any of the rings to skew the activity more specifically toward a certain AMPAR-auxiliary subunit complex. VU0539491 gave conflicting results between the VSD and calcium flux assay. It appears to be a PAM in A2R-stg and NAM in A2R-C3 cell lines but acts as a NAM in the A2Q-stg and A2Q cell lines and as a PAM in A2Q-GSG (Fig 6C). VU0539491 showed similar potency in all cell lines, but the compound is most efficacious on A2R-stg cells, as a PAM, in the raw data at ~2 μM. The glutamate potency fold-shift assay also identified it as a NAM on A2Q-stg and A2Q-C3 cells (Fig 5). This compound will be interesting to study because it can act as a PAM or a NAM depending on which auxiliary subunit is present.

We acknowledge that full auxiliary subunit occupancy cannot be guaranteed with most of our cell lines and low auxiliary subunit occupancy of the AMPAR may have masked less robust candidate hits. Also, the potencies reported herein may not reflect the potency of these compounds on a fully occupied receptor. We do, however, use a tethered construct for the A2Q-stg cell line, guaranteeing a fully occupied receptor which shows the same response as A2R-stg cells for VU0612951 and VU0627849.

Further characterization using electrophysiology to validate selectivity and potency is necessary for these hits. Fluorescent signal based assays do not measure the fast kinetics of the AMPAR. Specifically, while a deviation in GLUslope and AUC are good indicators that the compounds are acting as PAMs or NAMs, we are not directly measuring gating, which occurs in several milliseconds. An initial investigation of the compound VU0627849 by fast ligand application electrophysiology recapitulated the results obtained from the VSD assay. Confirming the specificity of these compounds or further tailoring these scaffolds to make them specific may offer a whole new class of compounds for basic research and clinical use. Differential expression patterns for auxiliary subunits throughout the central nervous system seem to be a naturally designed way to specifically target AMPARs in certain regions of the brain or times of development.

## Supporting information

S1 Table Primary VSD screen Analysis. Calculations from the primary VSD screen against A2R-stg cells. Each row represents a single well and the values that were calculated from the raw FDSS data using the Vanderbilt screening analysis platform WaveGuide. Each column represents a parameter calculation or sample description with detailed descriptions in row 2.
(XLSX)

S2 Table VSD counter-screens Analysis. Calculations from the VSD counter-screens against A2R-stg, A2R-C3, A2R and TetON cells. Each row represents a single well and the values that were calculated from the raw FDSS data using the Vanderbilt screening analysis platform WaveGuide. Each column represents a parameter calculation or sample description with detailed descriptions in row 2.
(XLSX)

S3 Table VSD known ligand CRC Analysis. Calculations from the VSD CRC assays for the known ligands fluorowillardiine, CX-546, and CTZ. Each row represents a single well and the values that were calculated from the raw FDSS data using the Vanderbilt screening analysis platform WaveGuide. Each column represents a parameter calculation or sample description with detailed descriptions in row 2.
(XLSX)

S4 Table Raw data for known ligand VSD CRC graphs. Raw FDSS data used to create graphs in Fig 2. Each sheet is labeled with a barcode that corresponds to a certain cell line. Cell line barcodes and ligand well assignments can be found in S3 Table, columns B, C, and E. Each row represents the Ex480/Em540 fluorescence values for a single well and each column from E to ON is a time point taken at 1Hz sampling.
(XLSX)

S5 Table VSD compound CRC Analysis. Calculations from the VSD compound CRCs against A2R-stg and A2R-C3 cells. Each row represents a single well and the values that were calculated from the raw FDSS data using the Vanderbilt screening analysis platform WaveGuide. Each column represents a parameter calculation or sample description with detailed descriptions in row 2. Highlighted columns were used to plot CRC graphs.
(XLSX)

S6 Table Calcium flux glutamate potency fold-shift Analysis. Calculations from the calcium flux glutamate potency fold-shift assay on A2Q-stg and A2Q-C3 cells. Each row represents a single well and the values that were calculated from the raw FDSS data using the Vanderbilt screening analysis platform WaveGuide. Each column represents a parameter calculation or sample description with detailed descriptions in row 2. Highlighted columns were used to plot glutamate CRC graphs and derive the values in Fig 5.
(XLSX)

S7 Table Calcium flux compound CRC Analysis. Calculations from the calcium flux compound CRCs against A2Q-stg, A2Q-C3, A2Q-GSG, A2Q cells. Each row represents a single well and the values that were calculated from the raw FDSS data using the Vanderbilt screening analysis platform WaveGuide. Each column represents a parameter calculation or sample description with detailed descriptions in row 2. Highlighted columns were used to plot CRC graphs.
(XLSX)

S8 Table Raw data for calcium flux compound CRC graphs. Raw FDSS data used to create graphs in Figs 7–9. Each sheet is labeled with a barcode that corresponds to a certain cell line. Cell line barcodes and VUcompound well assignments can be found in S7 Table, columns A, B, and D. Each row represents the Ex480/Em540 fluorescence values for a single well and each column from E to KR is a time point taken at 1Hz sampling.
(XLSX)
